# Late diagnosis of lumbar lipomyelomeningocele with tethered cord syndrome in an adult: case report and literature review

**DOI:** 10.1007/s00381-026-07232-w

**Published:** 2026-05-13

**Authors:** Milena Vieira Ramos, Bárbara A. Morais, Jairo Porfírio de Oliveira Júnior, Cilmaria Leite Franco, Nayara Mattos Pereira, Paulo Ronaldo Jubé-Ribeiro

**Affiliations:** 1 Division of Neurosurgery, Department of Surgery, Clinics Hospital, Federal University of Goiás, Goiânia, Goiás, Brazil; 2 Department of Neurological Surgery, Children’s Hospital, Goiânia, Goiás, Brazil; 3 Faculty of Medicine, University of Rio Verde, Goiânia, Goiás, Brazil

**Keywords:** Lipomyelomeningocele, Adult, Occult spinal dysraphism, Tethered cord syndrome, Case report

## Abstract

**Background:**

Lipomyelomeningocele (LMM) is the most common form of closed spinal dysraphism and is typically diagnosed in childhood. Although many patients are initially asymptomatic, progressive neurological deterioration may occur over time due to tethered cord syndrome (TCS). Adult presentation of untreated LMM is rare and poorly described in the literature, and optimal management strategies in this population remain controversial.

**Case:**

We report the case of a 53-year-old man with a congenital lumbosacral mass and a history of partial superficial lipoma resection in childhood, who presented with progressive sphincter dysfunction, lumbar pain, sexual dysfunction, and neurological decline. His clinical course was marked by long-standing unrecognized neurological impairment, leading to chronic lower extremity ulcerations, recurrent osteomyelitis, and multiple amputations. Magnetic resonance imaging demonstrated a large intra and extradural lumbar lipomyelomeningocele associated with tethered cord syndrome. The patient underwent microsurgical near-total resection of the intradural lipoma, detethering of the neural placode, dorsal neurulation, and expansile duraplasty. Postoperatively, he experienced significant clinical improvement, including recovery of spontaneous urination, reduced need for intermittent catheterization, resolution of neuropathic pain, and improvement in sexual function, without surgical complications.

**Conclusion:**

This case highlights the potential for severe and irreversible morbidity associated with delayed diagnosis and inadequate long-standing follow-up of lipomyelomeningocele. Although prophylactic surgery remains controversial, timely recognition, close surveillance, and individualized surgical intervention when symptoms arise may improve neurological and functional outcomes in adult patients. Multidisciplinary, long-term follow-up is essential to reduce morbidity and optimize quality of life in this rare but challenging population.

## Introduction

Lipomyelomeningocele (LMM) is the most common form of closed spinal dysraphism. It is characterized by a subcutaneous lipoma extending through a defect in the spine and attaching to an intradural mass, most commonly located in the lumbosacral region [1–4]. The condition is frequently diagnosed in childhood or early adulthood [5]. Approximately half of affected children present with a normal neurological examination during early life [2, 3].

A common and clinically significant complication of LMM is tethered cord syndrome (TCS), which may necessitate surgical intervention due to progressive neurological deterioration and involvement of urinary and bowel sphincter function [3, 6, 7].

Longitudinal studies have shown that up to 40% of patients experience clinical deterioration within 10 years, leading to the onset of symptoms and functional impairments [8]. In young adults, diagnosis is often delayed due to the insidious development of symptoms such as chronic back pain and lower extremity weakness, secondary to neural tethering [3, 5, 6].

There remains significant controversy regarding the indications for surgical treatment. The role of prophylactic surgery is particularly debated. Some authors advocate for early surgical intervention, arguing that neurological decline is inevitable and frequently irreversible once symptoms manifest [4, 9, 10]. Conversely, others support a conservative approach, citing the unclear natural history of the disease and the potential risks associated with surgery existing [11–13].

Importantly, most of the literature and clinical debate focuses on the pediatric population. The natural progression of LMM in adults who have not undergone surgical treatment remains poorly understood.

## Case report

**Fig. 1:**
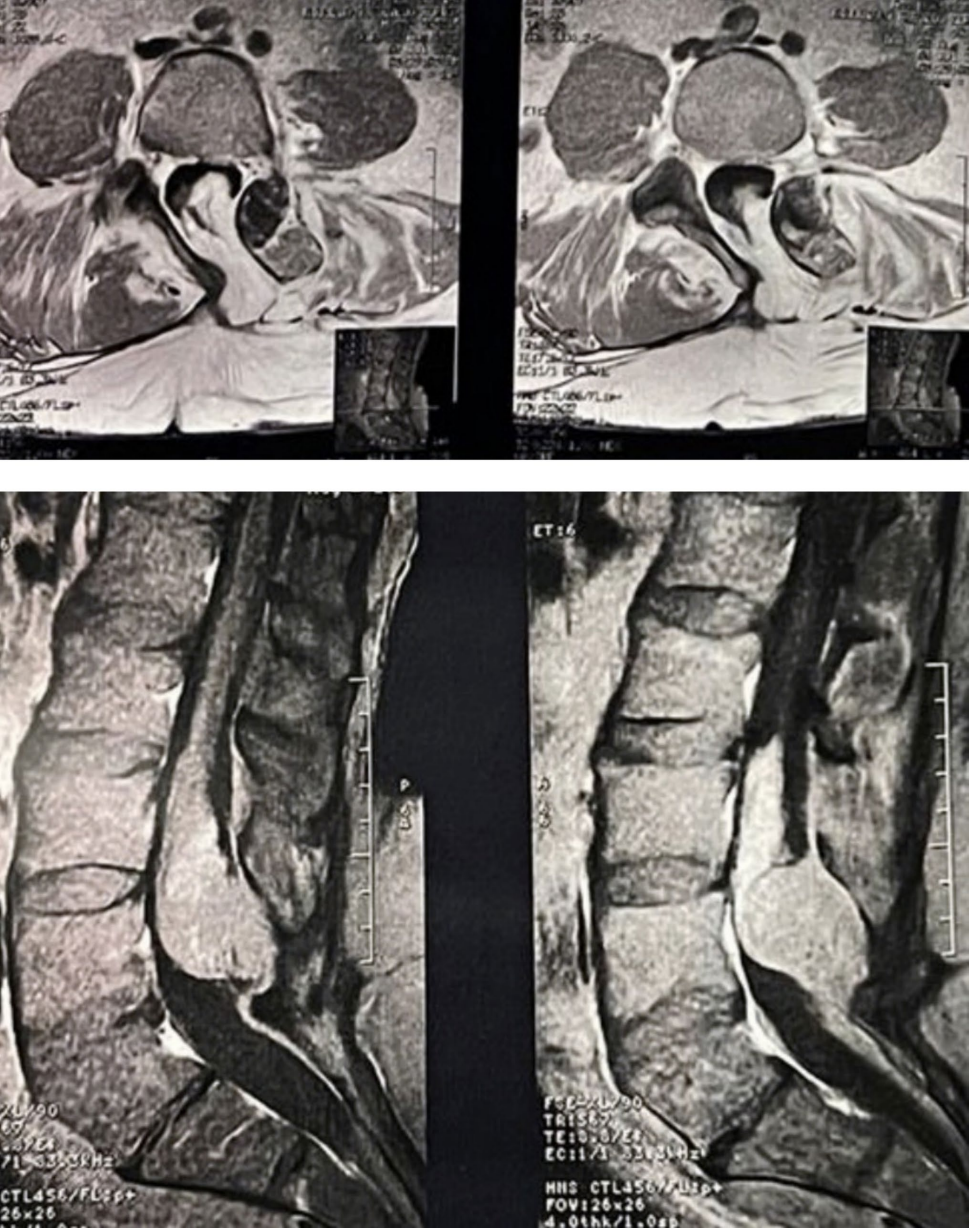
Lumbar spine MRI—preoperative evaluation

A 53-year-old male patient presented to the outpatient clinic with complaints of progressive loss of sphincter control. The patient reported a congenital dorsal lumbosacral swelling (Fig. 1) and had undergone partial resection of a superficial lipoma at the age of 7 at an outside institution.

At age 20, he developed chronic, non-healing ulcerative lesions on the left foot, associated with recurrent episodes of osteomyelitis. This led to multiple staged amputations, culminating in left lower limb amputation at age 27. Similar lesions appeared on the right foot, resulting in toe amputations at age 49, also secondary to chronic osteomyelitis. The patient denied any history of diabetes mellitus or tobacco use.

Despite these comorbidities, the patient remained ambulatory and functionally independent, using a prosthesis on the left leg. Nine months prior to presentation, he developed urinary retention and episodes of overflow incontinence. Urodynamic studies revealed a neurogenic bladder with diminished detrusor muscle contractile function. Intermittent bladder catheterization was initiated six times daily. In the subsequent months, he experienced worsening symptoms, including lumbar pain, fecal incontinence, and sexual dysfunction.

### Radiologic assessment

**Fig. 2:**
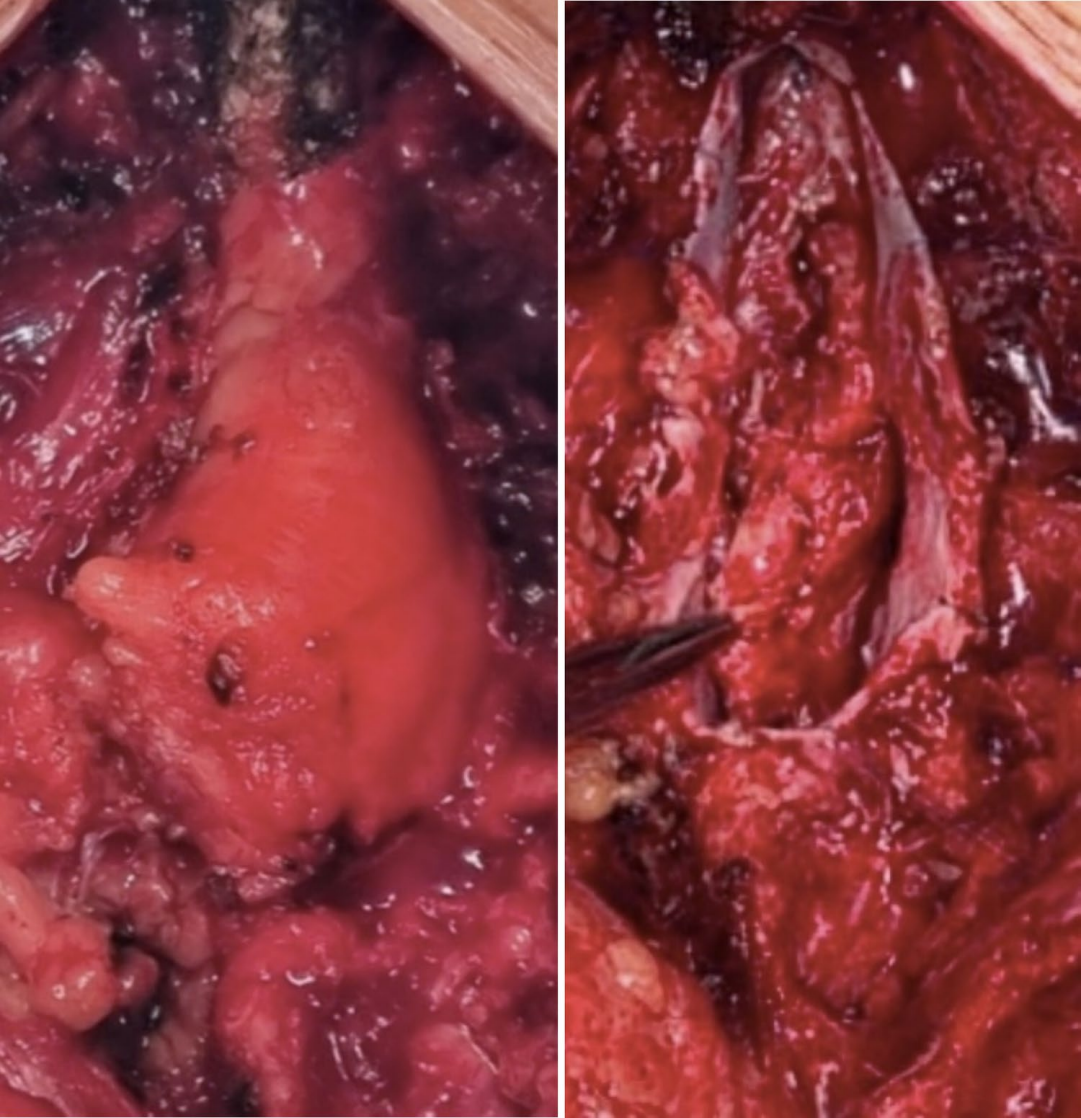
Surgical resection of the lipomeningocele

CT scan of the lumbosacral spine demonstrated posterior bone defects at L4-S1 vertebrae (Fig. 2).

Lumbar spine MRI revealed a large, heterogeneous lipomatous mass with intra and extradural components, causing compression of the neural elements. MRI of the entire neuroaxis demonstrated no other abnormalities (Fig. 2).

### Surgical intervention

**Fig. 3:**
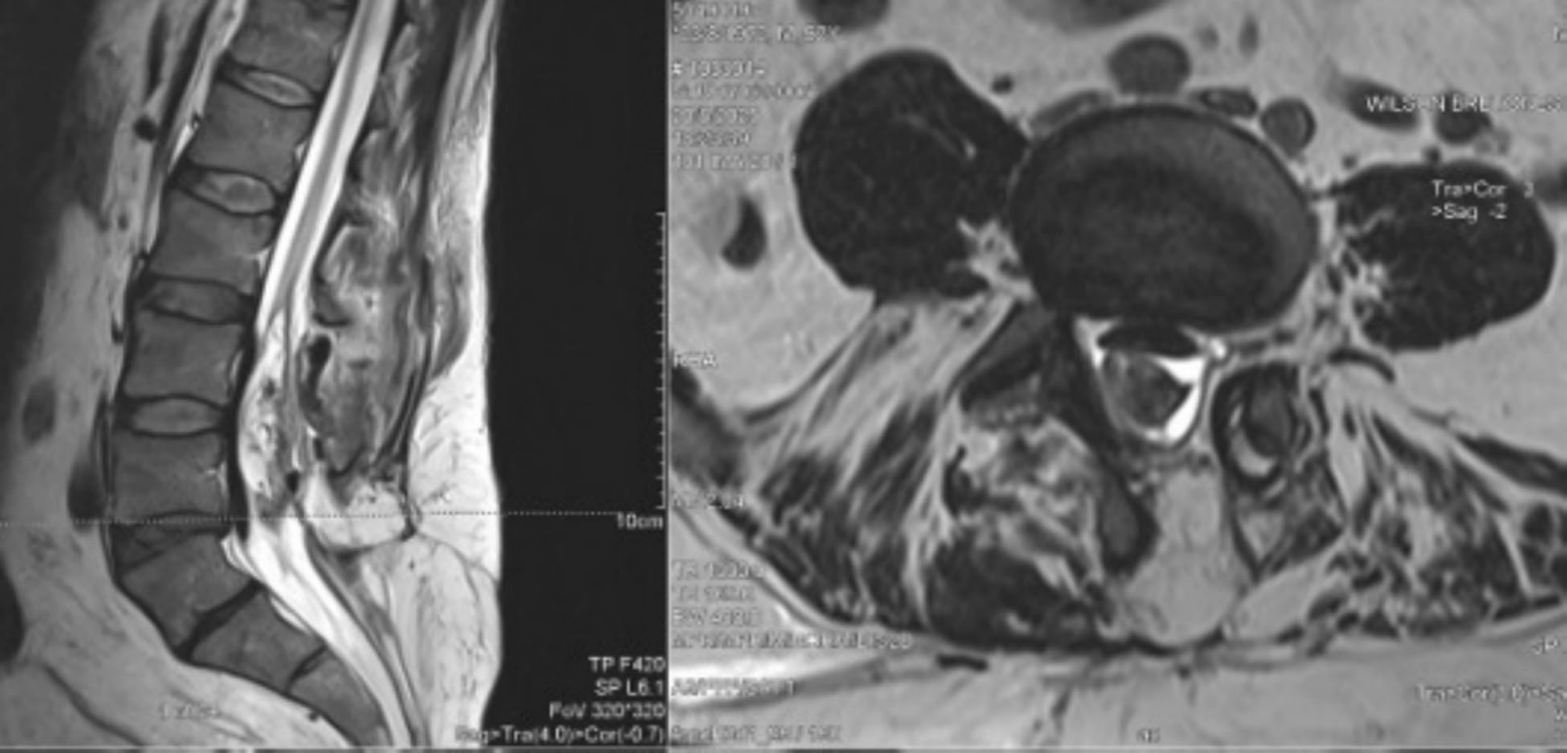
Lumbar spine MRI—postoperative evaluation

The patient underwent microsurgical treatment, consisting of near-total resection of the intradural lipoma. The procedure involved complete detachment of the lipoma and neural placode from the dura, pia-to-pia dorsal neurulation with microsutures, and expansile duraplasty using durapatch (Fig. 3).

### Postoperative course

The patient had an uneventful postoperative recovery. No cerebrospinal fluid leakage, wound complications, or neurological deterioration were observed. Postoperative MRI of the lumbar spine demonstrated extensive resection of the lesion with residual lipomatous components (Fig. 3). At 1-month follow-up, he revealed significant clinical improvement, including the return of spontaneous diuresis with reduced need for intermittent catheterization, resolution of preoperative burning neuropathic pain, and improvement in sexual function.

He continues in regular outpatient follow-up, with stable neurological status and no evidence of recurrent complications.

## Discussion

Adult presentation of lipomyelomeningocele (LMM) is uncommon; however, when previously reported cases are systematically compared, consistent patterns emerge that clarify the mechanisms underlying delayed diagnosis, neurological progression, and surgical outcomes. The structured analysis of the studies summarized in Table 1 demonstrates that late presentation is rarely incidental. Instead, it reflects a combination of prolonged compensated tethering, subtle early manifestations, absence of long-term surveillance, and, in some cases, incomplete childhood surgical management.

**Table 1:** Structured analysis of the studies

Study	Study type	*N* (patients)	Male to female	Mean age (years)	Time of symptoms (years)	Follow-up (months)	Clinical presentation	Location of the injury	Diagnosis	Present surgical treatment	Previous surgical treatment	Outcomes	Complications
Abu-Bonsrah, 2016	Case report	1	0:1	19	5	8	Neck pain, subcutaneous lump, 4/5 strength in the left lower extremity, decreased vibration sensation in both legs and positive Babinski sign bilaterally	Cervical-thoracic (1)	LMM C6-T6	Cervicothoracic laminectomy with subtotal resection	Laminectomy and duroplasty; subtotal resection	Subtotal resection	Wheelchair-bound user, baclofen pump-dependent, lower back pain and poorly controlled pain
Duz, 2007	Retrospective	3	3:0	21	-	6–12	Back pain (2); leg pain (1); bladder dysfunction (2);	Lumbar (1); sacral (2)	Tethered cord syndrome + LMM (3); dermal sinus (1)	Detethering (3); M resection (2); DS resection (1)	-	Improved bladder dysfunction, back pain (1)	-
Gürkanlar, 2007	Case report	1	1:0	22	-	60	No = no neurologic deficit (1)	Cervical (1)	LMM revealed C5-6 posterior fusion defect	Correction of LMM	-	No = no neurologic deficit (1)	-
Harazeen, 2022	Case report	1	1:0	19	1	-	Low back pain, urinary incontinence	Lumbar (1)	LMM + TCS (1)	TCS release surgery,lipoma excision	-	Low back pain, urinary incontinence	-
Krisht, 2015	Case report	1	0:1	58	2	12	3/5 strength in her left leg, 4/5 strength in her right, globally diminished sensation to light touch, and pin prick, as well as some bladder urgency, lower back pain	Lumbar (1)	Cord tethering, with an L4/L5 LMM extending to the sacral hiatus, Spinal dural arteriovenous fistulas (DAVFs)	Onyx embolization of feeding right and left lateral sacral arteries, 6 mon later LMM exploration/resection, DAVF ligation, and cord detethering	L4 and L5 laminectomy	5/5 strength throughout and 5/5 and 4/5 strength in her distal right and left lower extremities	DAVF extended into the lipomyelomeningocele with dilated draining veins rostrally and spinal canal involvement requiring a second surgical procedure after 6 months
North, 2019	Case report	1	0:1	45	40	12	Lumbar pain, neurogenic bowel and bladder, colostomy and urostomy, difficulty walking	Lumbarsacral (1)	LMM L2-S5, symptomatic hydrocephalus	Resection and closure of the meningocele	-	Transient paresthesia and mild weakness, baseline right lower extremity weakness and bilateral lower extremity sensory loss	-
Pang, 1982	Retrospective	23	10:13	39	33	6–132	Pain (18); sensoriomotor deficits (15); bladder dysfunction (13); bower dysfunction (5); trophic ulceration (3); rapid leg atrophy (2); increasing scoliosis (1); cutaneous manifestations (10)	Lumbosacral (23)	Tethered cord syndrome (23)	Release of tethered conus (22)	-	Relief of pain (15); sensoriomotor deficits (12); sphincter dysfunction (13); trophic ulceration (3); scoliosis (2); pes cavus (5)	Death (1)
Ramos, 2026	Case report	1	1:0	53	33	1	Sphincter dysfunction, lumbar pain, sexual dysfunction, neurological decline	Lumbosacral (1)	Intradural lipoma	Near total resection	Partial resection, amputations	Spontaneous urination, pain resolution, improve sexual function	None

Across the reviewed cases, symptom duration prior to definitive intervention frequently spanned decades. In Pang’s adult series, the mean age at presentation was 39 years with an average symptom duration of 33 years, while North described a patient who experienced progressive deterioration for 40 years before surgery. Similarly, in our case, diagnosis occurred at 53 years of age after more than three decades of progressive neurological impairment. These observations reinforce the concept that tethered cord physiology may remain partially compensated for extended periods before cumulative mechanical traction and ischemic stress result in irreversible neural injury. The relative mildness of early symptoms likely contributes to diagnostic inertia and loss of follow-up during the transition from pediatric to adult care.

Pain consistently represents the most frequent and often earliest manifestation across adult reports. Lumbar or radicular pain was a dominant complaint in Pang’s series and in smaller contemporary reports, including Düz, Harazeen, and North. Although pain may prompt evaluation, it does not necessarily indicate advanced neurological compromise. In contrast, bladder dysfunction appears to correlate more strongly with disease progression. In Pang’s cohort, more than half of patients presented with sphincter impairment, and additional reports documented neurogenic bladder involvement in symptomatic adults. In our patient, progressive urinary retention and overflow incontinence were decisive factors leading to neurosurgical referral, suggesting that sphincter dysfunction may represent a late but clinically decisive stage of tethered cord decompensation.

Motor deficits were variable among studies. Some patients exhibited mild weakness that improved after surgery, whereas others remained significantly disabled despite intervention. Notably, one reported adult patient remained neurologically intact, demonstrating that anatomical LMM does not inevitably produce early deficits. This heterogeneity highlights the unpredictable tempo of progression but also suggests that once motor or sphincter deficits become established and prolonged, reversibility diminishes.

Previous incomplete surgical treatment appears to be an important contributor to late deterioration. Cases described by Abu-Bonsrah and Krisht involved prior procedures before definitive detethering, and persistent or progressive deficits were observed in some of these patients. In our case, childhood management consisted only of superficial lipoma excision without formal detethering. The persistence of tethering forces likely facilitated chronic neurological decline, ultimately culminating not only in sphincter dysfunction but also in systemic complications such as recurrent ulcerations, osteomyelitis, and amputations. These findings suggest that incomplete early intervention may predispose patients to long-term morbidity by allowing ongoing mechanical traction of neural elements.

When surgical outcomes are examined comparatively, symptom reversibility appears domain-specific. Pain demonstrates the highest likelihood of improvement, with substantial relief reported in multiple series. Motor recovery is more variable and appears closely related to the duration and severity of preoperative deficits. Sphincter dysfunction shows the least predictable reversibility; although improvement was documented in some cohorts, persistent or worsened bladder dysfunction was also observed, particularly in long-standing cases. In our patient, near-total resection with detethering resulted in recovery of spontaneous urination, reduction in catheterization frequency, resolution of neuropathic pain, and improvement in sexual function. This outcome illustrates that meaningful functional gains remain achievable even in delayed adult presentations, although complete neurological restitution should not be anticipated.

Surgical morbidity, while not negligible, was generally acceptable in symptomatic patients. Complications reported across series included death, need for reoperation, and persistent neurological deficits. These findings underscore that surgical indication must carefully balance the potential for neurological improvement against procedural risks, particularly in asymptomatic or minimally symptomatic individuals.

The controversy surrounding prophylactic surgery remains unresolved. The comparative analysis suggests that not all anatomically diagnosed LMMs progress rapidly, as evidenced by neurologically intact adults. However, a substantial proportion of patients with prolonged follow-up eventually develop progressive pain, motor decline, or bladder dysfunction. The aggregated data therefore support a risk-adapted rather than universal approach. Patients demonstrating emerging sphincter dysfunction, progressive motor deficits, or prior incomplete surgical management may represent subgroups in whom earlier surgical consideration is justified to prevent irreversible neurological compromise. Conversely, careful longitudinal surveillance may be reasonable in truly asymptomatic individuals.

Finally, our case expands the discussion beyond neurological parameters. The long-standing untreated tethering in this patient resulted not only in progressive neurological decline but also in severe systemic sequelae, including chronic ulcerations, recurrent infections, and limb amputations. This observation highlights that delayed diagnosis carries substantial functional and socioeconomic consequences and reinforces the importance of structured long-term follow-up.

In summary, the comparative review of adult LMM cases demonstrates that delayed presentation commonly reflects decades of compensated tethering; pain is the most reversible postoperative symptom; motor recovery is inversely related to duration of deficit; and long-standing sphincter dysfunction carries a guarded prognosis. Repeated or incomplete surgical interventions may increase cumulative morbidity. Therefore, individualized, progression-guided surgical decision-making combined with structured long-term surveillance appears central to optimizing outcomes in adult patients with lipomyelomeningocele.

## Conclusion

Adult presentation of lipomyelomeningocele represents the late clinical expression of a chronically tethered spinal cord that may remain partially compensated for decades. The comparative analysis of previously reported cases demonstrates that delayed diagnosis is commonly associated with subtle early symptoms, absence of structured long-term follow-up, and, in some instances, incomplete childhood surgical treatment. Pain is the most frequent and most reversible postoperative symptom, whereas motor deficits and long-standing sphincter dysfunction carry a less predictable prognosis, particularly when symptom duration is prolonged.

The aggregated evidence does not support a universal prophylactic surgical strategy; however, it suggests that selected subgroups—particularly patients with emerging bladder dysfunction, progressive motor decline, or prior incomplete detethering—may benefit from earlier intervention to prevent irreversible neurological compromise. Conversely, asymptomatic individuals may be managed with careful longitudinal surveillance.

Ultimately, optimal management of adult lipomyelomeningocele requires individualized, risk-adapted decision-making grounded in clinical progression rather than anatomical diagnosis alone. Structured long-term follow-up from childhood into adulthood remains essential to mitigate the substantial neurological and systemic morbidity associated with delayed recognition and treatment.

A coordinated multidisciplinary approach, integrating neurosurgical, urological, and rehabilitative expertise, remains fundamental to minimizing cumulative morbidity, preserving functional autonomy, and optimizing long-term outcomes in adults with lipomyelomeningocele.

## Data Availability

No datasets were generated or analysed during the current study.
